# Supplementary material for: Risk factors for eight common cancers revealed from a phenome-wide Mendelian randomisation analysis of 378,142 cases and 485,715 controls
Source: Res Sq. 2023 Mar 17:rs.3.rs-2587058. Preprint. [Version 1] doi: 10.21203/rs.3.rs-2587058/v1 (PMC10055507; doi:10.21203/rs.3.rs-2587058/v1)
Supplement: 1 [file NIHPPrs2587058v1-supplement-1.pdf]

SUPPLEMENTARY TABLES LEGENDS

Supplementary Table 1. List of traits examined in the Mendelian randomisation analysis and estimate of power for each trait and cancer type.

Supplementary Table 2. Effect allele, frequency, effect on trait and strength of association with each cancer type for SNPs used as instrumental variables.

Supplementary Table 3. Causal estimates from the Mendelian randomisation analysis for continuous traits and cancer risk.

Supplementary Table 4. Causal estimates from the Mendelian randomisation analysis for continuous traits and breast cancer subtype.

Supplementary Table 5. Causal estimates from the Mendelian randomisation analysis for continuous traits and lung cancer subtype.

Supplementary Table 6. Causal estimates from the Mendelian randomisation analysis for continuous traits and ovarian cancer subtype.

Supplementary Table 7. Weighted median estimate and mode-based estimates for each trait and cancer type.

Supplementary Table 8. MR-Egger regression analysis for each trait and cancer type.

Supplementary Table 9. MR Steiger analysis for each continuous trait and cancer type.

Supplementary Table 10. Lifetime risk of each cancer used to calculate the proportion of variance explained.

Supplementary Table 11. Leave one out inverse variance weighted random-effects MR analysis for each exposure trait and cancer type.

Supplementary Table 12. The hierarchical levels of statistical support used to classify associations.

Supplementary Table 13. Causal estimates for each Mendelian randomisation method for each binary trait and cancer risk.

Supplementary Table 14. Causal estimates for each Mendelian randomisation method for each binary trait and breast cancer subtype.

Supplementary Table 15. Causal estimates for each Mendelian randomisation method for each binary trait and lung cancer subtype.

Supplementary Table 16. Causal estimates for each Mendelian randomisation method for each binary trait and ovarian cancer subtype.

Supplementary Table 17. Details of filtering applied to instrumental variables used in the Mendelian randomisation analysis.

Supplementary Table 18. Literature triples identified across eight different cancer types and Mendelian randomisation defined risk factors using SemMedDB.

Supplementary Table 19. Stratification of literature space size by trait category.

Supplementary Table 20. List of potential mediators for each trait identified using SemMedDB.
